# Cutaneous Vasculitis, Interstitial Pneumonia with Crazy-Paving Appearance, and Positive pANCA in a Patient with Severe Crohn's Disease

**DOI:** 10.1155/2014/485714

**Published:** 2014-08-14

**Authors:** Guang-liang Chen, Juan Wang, Bao-zhen Li, Li-mei Li, Han-you Mo, Shuang Ye

**Affiliations:** ^1^Department of Rheumatology & Immunology, Affiliated Hospital of Guilin Medical University, 5th Floor, Building No. 3, 15 Lequn Road, Guilin 541001, China; ^2^Department of Internal Medicine 3, University of Erlangen-Nuremberg, 91054 Erlangen, Germany; ^3^Department of Rheumatology, Huashan Hospital Affiliated to Fudan University, Shanghai 200040, China; ^4^Department of Rheumatology, Renji Hospital Affiliated to Shanghai Jiao Tong University School of Medicine, 145 Shandong C Road, Shanghai 200001, China

## Abstract

Cutaneous vasculitis, interstitial pneumonia with crazy-paving appearance on high-resolution computed tomography, and repeated positive perinuclear anti-neutrophil cytoplasmic antibodies (pANCA) are rarely found together in patients with inflammatory bowel disease in the existing literature. We report the case of a Chinese patient previously diagnosed with cutaneous vasculitis and interstitial pneumonia, who presented with acute pain and mass in his right lower quadrant a couple of years later. The terminal ileum biopsy and postoperative pathology confirmed Crohn's disease (CD).

## 1. Introduction

Extraintestinal manifestations of Crohn's disease (CD) are prevalent [1–5]. However, the diversity and nonspecific manifestations can be a challenge to physicians in managing this disease. Usually, these manifestations occur in the late course of CD and involve nearly any organ system, including the dermatologic and pulmonary systems [2–7]. It is seldom reported that extraintestinal manifestation is the first symptom of CD. We, herein, report a Chinese patient with CD, whose cutaneous vasculitis concomitant with pulmonary manifestation antedates the onset of CD by approximately 3 years. Interestingly, the pulmonary manifestation presented as interstitial pneumonia with a crazy-paving appearance (CPA) on high-resolution computed tomography (HRCT) in the lungs.

## 2. Case Presentation

**Figure 1:**
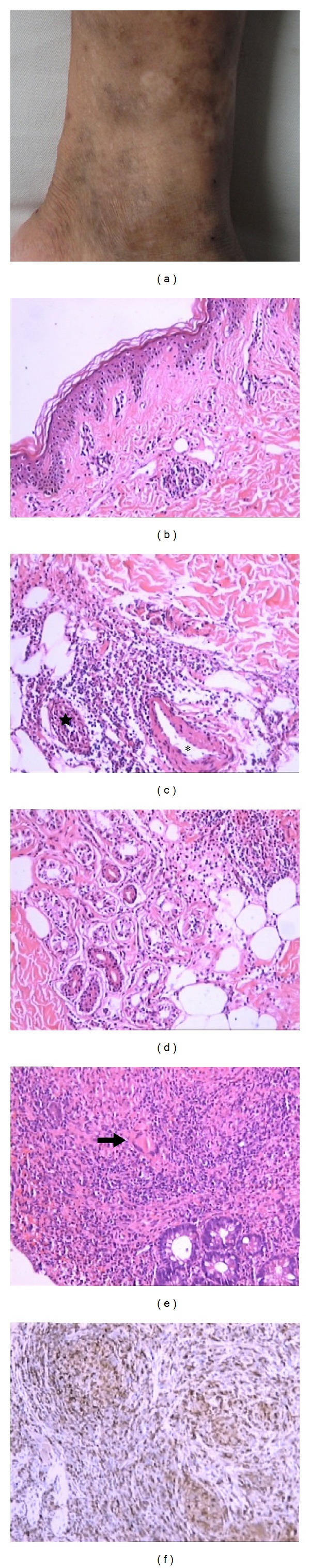
Skin lesions and pathologic findings. (a) A representative picture for skin nodules. ((b)–(d)) Histological examination of skin lesions showed mild atrophy of the epidermis and enormous neutrophilic and lymphocytic infiltration of adipose tissue and appendages in the dermal layer ((b), (d)); local small abscesses were formed (star) (c); in line with vasculitis, a thickening of the arterial walls of some small arteries by an inflammatory lymphoid infiltrate (snowflake) (H.E. stain ×100) (c). ((e)-(f)) Biopsy from the terminal ileum mucosa showed a granulomatous inflammatory infiltration (black bold arrow) (H.E. stain ×100) (e). Antiacid staining and PAM were negative. CD68 antigen in terminal ileum mucosa was positive (×100) (f).

**Figure 2:**
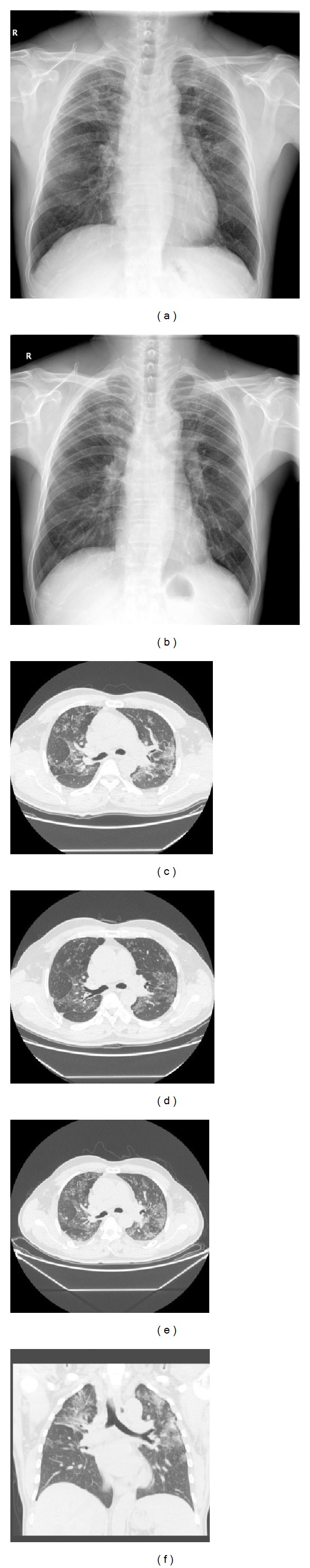
X-ray films of the chest were taken in June 2010 (a) and September 2012 (b). High-resolution computed tomography (HRCT) showed diffuse ground-glass opacities and a crazy-paving appearance in both lungs. (c), (d), and (e) were taken in July 2010, December 2010, and June 2011, respectively. (f), which was taken in June 2011, showed the lesions in both of the upper lungs.

A 55-year-old Chinese male developed recurrent skin eruptions, which were tender and pruritus and lasted approximately 1 year. Skin lesions (Figure 1(a)) appeared around the entire body, especially on the lower extremities, which presented as ulcerations and healed slowly with pigmentation. He had nonproductive cough and severe dyspnea on exertion and was initially admitted to a local primary hospital in May 2010. The chest X-ray showed patchy and diffuse interstitial infiltration in both lungs (Figure 2(a)). The patient was transferred to our department in June 2010.

The medical history included chronic varicosity on the left lower extremity with 30 years of duration and enlargement of axillar and inguinal lymph nodes for 2 years. He was a nonsmoker and had a job in poultry cultivation since he was young. Elevated erythrocyte sedimentation rate (ESR, 0–15 mm/h) of 60 mm in the first hour, c-reactive protein (CRP, ≤1 mg/dL) of 10.17 mg/dL, serum lgG (8–16 g/L) of 39.3 g/L, serum globulin (25–30 g/L) of 42.82 g/L, and thyroid-stimulating hormone (TSH, 0.7–6.4 *μ*IU/mL) of 6.4 IU/mL were appreciated, while the blood cell count, urine analysis, and the rest of the biochemical studies were normal. Assays including human immunodeficiency virus (HIV), hepatitis A virus (HAV), hepatitis B virus (HBV), hepatitis C virus (HCV), syphilis serology (RPR), purified protein derivative (PPD), and tumor markers were negative. Serodiagnostics for* Clonorchis sinensis* and* Cysticercus cellulosae* were also negative. Anti-cardiolipin antibodies (ACL-lgG/lgM) and pANCA (determined by enzyme-linked immunosorbent assay (ELISA)) were positive. Anti-nuclear antibody (ANA) was a borderline positive in a titre of 1 : 100, while ENA and complement were normal. HRCT of the chest showed diffuse ground-glass opacities and a crazy-paving appearance (CPA) (Figures 2(c)–2(f)). Abdominal ultrasound showed mild enlargement in the spleen (47 mm thickness). Electrocardiogram was unremarkable. Biopsy from one of the skin lesions and enlarged inguinal lymph nodes revealed nonspecific vasculitis (Figures 1(b)–1(d)) and lymphadenitis, respectively.

He was diagnosed with cutaneous vasculitis and interstitial pneumonia. He was treated with corticosteroid (40 mg i.v. methylprednisolone) and cyclophosphamide (CYC; 0.6 g~1.2 g was administered intravenously once a month for 6 months and subsequently once every 3 months). Thalidomide (50 mg/day) was also added orally. Skin eruptions resolved and the symptom of dyspnea improved in parallel with radiographic changes (Figure 2(d)). In September 2010, after a herpes zoster infection, the steroid dose was reduced to 7.5 mg/day. Since the cumulative dose had reached 12 g in December 2011, CYC was discontinued and hydroxychloroquine (HCQ) (400 mg/day) was initiated. However, no further improvement of the interstitial lung disease could be appreciated (Figures 2(e) and 2(f)).

**Figure 3:**
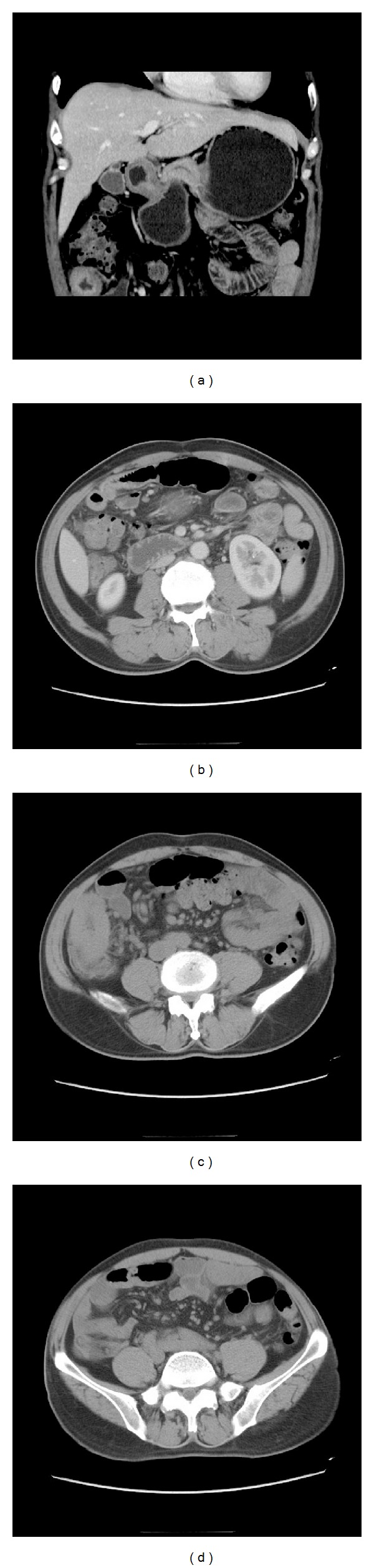
HRCT scan of the abdomen showed severe segmental narrowing, a cobblestone appearance of the terminal ileum, small-bowel wall thickening, and mesenteric adenopathy.

On January 3rd, 2012, the patient was admitted again for evaluation of two-week acute-onset abdominal pain. Physical examination revealed that the patient was hemodynamically stable and had middle-grade fever. A mild tenderness on the right lower quadrant was found. There was no progression in skin eruptions on the lower extremities. A HRCT scan of the abdomen showed severe segmental narrowing and a cobblestone appearance in the terminal ileum (Figure 3). Ulcerations in the mucous were revealed by colonoscopy. Biopsy from the mucous verified a granulomatous inflammatory infiltration (Figures 1(e) and 1(f)). The patient was empirically treated with a standard anti-TB therapy for nearly 3 months without improvement. A repeated colonoscopy was performed. The transverse colon mucosa near the hepatic flexure was swollen and had a nodular appearance. The pathologic finding showed severe inflammation and mucosal ulceration with a large number of lymphocytes and neutrophil infiltration but without granulomas. In the middle of April 2012, a third colonoscopy showed severe acute and chronic inflammation in the terminal ileum mucosa accompanied by chronic ulcerations (some areas presented as fissure-like lesions). Moreover, the acid-fast staining and PCR for* Mycobacterium tuberculosis* were all negative. Anti-TB treatment was stopped, and the patient was switched to methylprednisolone (MP) plus mesalazine. But he deteriorated with an abdominal intestinal fistula formation and lost nearly 20 kg weight in 3 months. A subtotal colectomy and ileostomy were performed in June 2012. The postoperative pathologic findings of the intestines showed pathologic changes typical of CD. The lymph nodes showed reactive mesangial hyperplasia and hyaline degeneration. Acid-fast staining, periodic acid-Schiff (PAS), and periodic acid methenamine (PAM) in the mucosa were negative. After the surgical treatment, he recovered gradually. The reexamination of the chest X-ray was also improved significantly (Figure 2(b)), while the pANCA testing was still strongly positive. The patient received corticosteroid (5 mg/day) and tripterygium glycosides (30 mg/day) as maintenance and remained stable in the subsequent 2-year follow-up.

## 3. Discussion

The coexistence of cutaneous vasculitis and interstitial lung disease in CD has never been reported. To the best of our knowledge, this is the first case indicating that the pulmonary manifestation in CD could present as interstitial pneumonia with a CPA on HRCT in the lungs.

Skin lesions occur in 14–44% of patients with CD [1]. However, as an initial symptom of CD, cutaneous vasculitis was seldom found in the published literature. Mader et al. [2] searched in the MEDLINE database and only one case presented with cutaneous leukocytoclastic vasculitis as the initial manifestation of CD 3 months before the bowel disease was reported. Similarly, Tsiamoulos et al. [3] reported a case of an octogenarian with cutaneous vasculitis in the skin as the first symptom of CD 2 months before the bowel disease. The typical pathology patterns of these CD-related cutaneous lesions include vasculitis, extravascular neutrophilia, and granulomatous inflammation [1]. In the present case, cutaneous vasculitis occurred 3 years prior to the bowel disease with relatively nonspecific vasculitic changes. However, the link between the cutaneous lesions and CD in this patient cannot be excluded. Consistent with the observations reported by Tsiamoulos et al. [3] and Zlatanic et al. [4], the skin lesions related to CD likely have a good response to the immunosuppressant therapy. And, in our case, the aggressive immunosuppressive treatment for cutaneous vasculitis and interstitial lung disease may in fact retard the subsequent development of full-blown CD.

Pulmonary involvement in CD has previously been reported with an estimated prevalence of 0.4% in a study of 1400 patients [5–7]. The airways and lung parenchyma were more commonly affected by granulomatous inflammation or nongranulomatous inflammation owing to the preexisting CD [5–7]. Shulimzon et al. [8] described two female patients with CD whose necrotizing granulomatous inflammation in the lung occurred 5 years before the bowel symptoms. Roblin et al. [9] also reported a pediatric case with granulomatous pulmonary involvement before the diagnosis of CD. In our case, pulmonary manifestation antedates the onset of CD by nearly 2 years with a CPA pattern found by HRCT. CPA has a variety of causes, including infectious, neoplastic, idiopathic, inhalational, and autoimmune disorders [10]. However, CPA has never been reported in CD patients. Despite the lack of open lung biopsy data, the observation of the symptoms and radiographic findings of the lungs subsided after the surgery suggested this connection. Indeed, it has been reported [11] in a case series that included seven CD patients where the disease activity in the pulmonary and intestine paralleled.

CD is characterized as a granulomatous systemic autoimmune disease, and 10%~20% of the patients presented with a variety of autoantibodies [12–14]. In 1996, Vasiliauskas et al. suggested that pANCA identifies a subset of CD with a UC- (ulcerative-colitis-) like presentation [13], but this cannot be validated in an independent cohort [14]. Here, the pANCA testing of the patient was repeatedly positive without a UC-like presentation, suggesting the presence of genetic heterogeneity in pANCA as a biomarker for the disease, which was also involved in the data shown by Prideaux et al. in 2013 [15]. However, serological markers seem to be unable to indicate disease activity according to the study by Rieder et al. [16]. Likewise, we showed that pANCA is stable during the course of the disease, indicating that pANCA is another such serological marker. In addition, there are several reports in patients with coexisting rheumatologic disorder and CD, such as Wegener's granulomatosis [17], SLE [18], and Takayasu's arteritis [19]. Nevertheless, the presence of pANCA in our case may explain the vasculitic components of the cutaneous lesion and interstitial lung disease, albeit a specific diagnosis of ANCA-associated vasculitis cannot be established.

## Abbreviations

ANCA: Anti-neutrophil cytoplasmic antibodies
ANA: Anti-nuclear antibody
ACL: Anti-cardiolipin antibodies
CRP: C-reactive protein
CD: Crohn's disease
CYC: Cyclophosphamide
ESR: Erythrocyte sedimentation rate
ELISA: Enzyme-linked immunosorbent assay
HIV: Human immunodeficiency virus
HAV: Hepatitis A virus
HBV: Hepatitis B virus
HCV: Hepatitis C virus
HRCT: High-resolution computed tomography
HCQ: Hydroxychloroquine
MP: Methylprednisolone
Lg: Immunoglobulin
RF: Rheumatoid factor
RPR: Rapid plasma reagin
PPD: Purified protein derivative
PAS: Periodic acid-Schiff
PAM: Periodic acid methenamine
PCR: Polymerase chain reaction
pANCA: Perinuclear anti-neutrophil cytoplasmic antibodies
TSH: Thyroid-stimulating hormone
UC: Ulcerative colitis.
